# A rare case of pituitary metastasis from hepatocellular carcinoma: case report and literature review

**DOI:** 10.3389/fonc.2023.1227678

**Published:** 2023-07-27

**Authors:** Yuanchen Cheng, Ming Feng

**Affiliations:** ^1^ Department of Neurosurgery, Peking Union Medical College Hospital, Chinese Academy of Medical Sciences and Peking Union Medical College, Beijing, China; ^2^ School of Medicine, Tsinghua University, Beijing, China

**Keywords:** pituitary, hepatocellular carcinoma, metastases, surgical treatment, pathology

## Abstract

Presence of pituitary metastases (PMs) is a relatively rare clinical situation, especially when originating from hepatocellular carcinoma (HCC). A 73-year-old man presented with headaches, diplopia, and soon impaired vision, as well as gastrointestinal symptoms. Computed tomography (CT) and magnetic resonance imaging (MRI) of the brain revealed a space-occupying mass in the sellar region. The patient had a history of hepatocellular carcinoma and recent abdominal ultrasound and positron emission tomography (PET) indicated recurrence and metastases. Endoscopic transnasal transsphenoidal tumor excision was performed, and postoperative pathological report confirmed the diagnosis of HCC PM. In the literature review, 17 published cases of HCC PMs were summarized. Both the diagnosis and management of HCC PMs are difficult. Patients who had HCC-related history and new-onset headaches or diplopia should be inspected with a suspicion of metastatic lesions. Surgical intervention with transnasal endoscope is only recommended to ameliorate the symptoms and improve the life quality.

## 1 Introduction

PMs are rare among all sellar lesions. The reported incidence of metastatic lesions among all surgically removed pituitary tumors is approximately 1% (1). However, the incidence in certain autopsy series is much higher (2), perhaps due to the possible asymptomatic nature of PMs. Many researchers agreed that diabetes insipidus (DI) is the most common symptom of PMs, which distinguishes PMs from pituitary adenomas (3). The posterior lobe of the pituitary is more vulnerable to metastases, possibly due to its direct arterial supply (4).

It has been reported that the primary cancer can originate from multiple sites (5, 6). Among all, breast and lung cancers are widely recognized as the two most prevalent origins, accounting for about 60% of all PMs (6). It is hypothesized that this preference is hormone-dependent, for example, the prolactin-rich environment in pituitary promotes the proliferation of breast cancer metastases (3). HCC remains one of the most common and fatal malignancies worldwide (7). Even though HCC is a relatively infrequent origin of PMs, its highly invasive and rapidly progressive nature made the diagnosis and management of HCC PMs extremely complicated. HCC PMs are seldom reported in the literature, with only 17 cases published so far. Here we reported another case of a 73-year-old male with a previous history of HCC presented with severe headaches and diplopia. The clinical characteristics of all reported cases were also summarized, in order to provide more insights into the diagnosis and management of HCC PMs.

## 2 Methods

### 2.1 Literature review

Pertinent literature was searched using Pubmed for English references and CNKI for Chinese references. Keywords “pituitary metastases” and “hepatocellular carcinoma” were searched in all fields. 23 English articles and 1 Chinese article were retrieved, of which 13 were unrelated and thus excluded. Careful reviews were executed in the rest of the articles, and 5 more references were identified from the bibliographies. Eventually, 17 reported cases of HCC PMs were included in this review.

### 2.2 Case report

A 73-year-old man with a history of severe headaches for 1 month, and diplopia for 2 weeks was admitted to the Department of Neurosurgery at Peking Union Medical College Hospital on May 10, 2022. Previously, local hospital made a diagnosis of left genyantritis and prescribed conservative analgesic medication. His headache attacks were mitigated after the medication. Two weeks later, with no precipitating factors, a sudden onset of diplopia and blurred vision on the right side was developed. He also had nausea, vomiting, and abdominal discomfort for six days.

On admission, blood pressure was 140/90mmHg, and other vital signs were normal (temperature: 36.5°C, heart rate: 64bpm, respiratory rate: 16/min). Neurological examination identified abducens nerve palsy with binocular cohesion and limited abduction. Visual acuity was recorded as 0.5 for left eye and 0.6 for right eye (converted as 20/63 and 20/80 in Snellen eye chart). A further visual field test revealed temporal field defects. Other ocular movements were intact and pupillary light reflexes were normal. Further neurological tests found that he was unable to perform the finger-to-nose test and the Romberg sign was probable positive.

**Figure 1:**
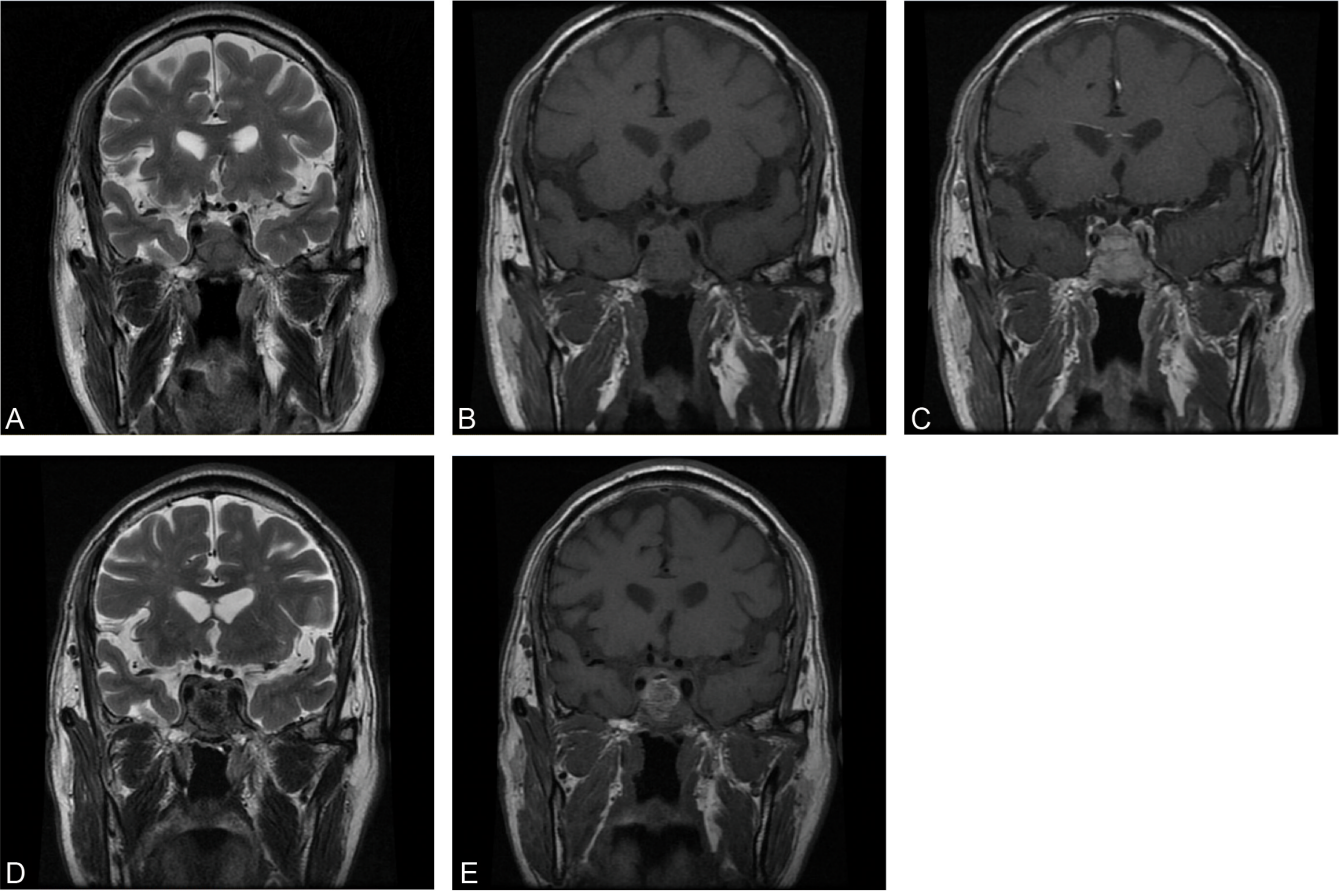
Preoperative coronal MRI of precontrast T2WI **(A)**, precontrast T1WI **(B)**, and enhanced T1WI **(C)**. Postoperative coronal MRI of precontrast T2WI **(D)** and precontrast T1WI **(E)**.

A CT brain scan showed a soft-tissue density mass in the sellar region and the sphenoid sinus, and bone density was decreased in the clivus. Subsequent enhanced MRI of the brain depicted a homogeneously enhancing pituitary mass extending into the bilateral sphenoid sinus and invading the clivus, measuring approximately 3.1×3.4×2.7 cm in size (**Figures 1A–C**). Endocrinological tests detected an elevation of cortisol level in both blood (28.2 µg/dl, normal range 4.0-22.3 µg/dl) and urine (24hUFC 245.1 µg/dl, normal range 12.3-103.5 µg/dl). Other basal endocrinology tests including growth hormone (GH), insulin-like factor 1 (IGF1), and thyroid hormones (including T3, T4, FT3, FT4, TSH) were all normal. Urea and electrocytes (including sodium, potassium, calcium, etc.) were within normal ranges.

The patient had a history of hepatitis B virus for more than 20 years and underwent surgical resection in 2014 and radiofrequency ablation in 2017 for HCC. Liver biochemical tests revealed no abnormality. Alpha-fetoprotein (AFP) was 92.2 ng/ml (normal level ≤20 ng/ml). Other tumor markers were all within normal limits. Abdominal ultrasound detected a 7.6×7.0 cm hypersonic mass in the right posterior section of the liver. PET highlighted the possibility of recurrence and metastasis of hepatocellular carcinoma, with increased radiation uptake in multiple sites including the liver, vertebral bodies, and costal bones. However, no abnormally elevated uptake was detected within the brain parenchyma.

**Figure 2:**
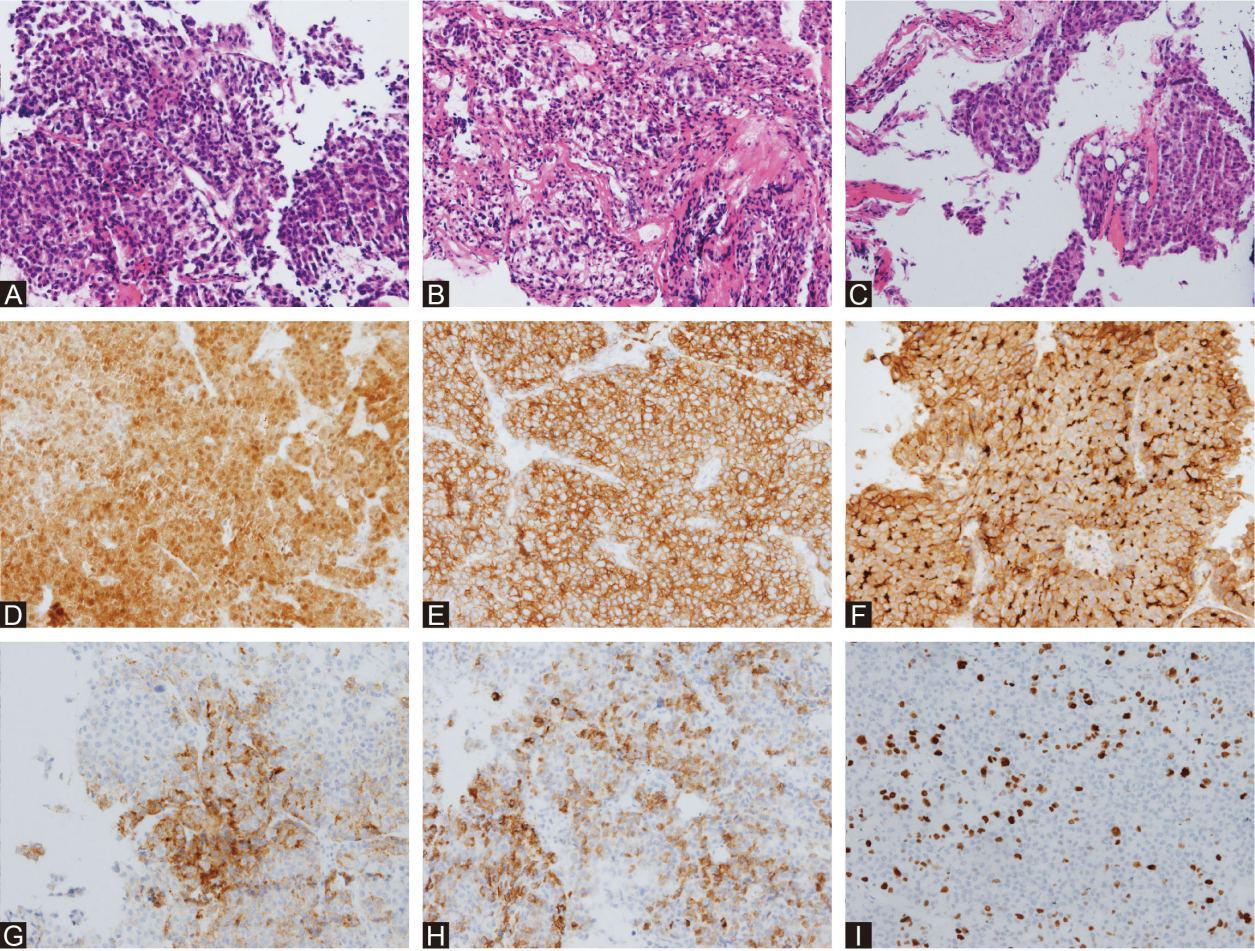
Histological surgical specimens. HE staining of the intrasellar mass **(A)** showed polymorphic cytological structure with a high nuclear-to-cytoplasmic ratio and an acinar to glandular growth pattern. HE staining of the dura mater **(B)** showed that the collagen fibers were irregular and widely invaded by tumor cells. HE staining of the saddle floor bone **(C)** showed that normal trabecular and cell patterns were largely interrupted by tumor cells. Immunohistochemical staining of Arg-1 **(D)**, CAM5 **(E)**, CD10 **(F)**, GPC-3 **(G)**, hepatocyte **(H)**, and Ki-67 **(I)** confirmed the diagnosis of metastatic HCC.

In combination with this patient’s history, neurological examination, and imaging findings, an initial inference of HCC PM was made. The diagnosis was made mainly based on his malignancy history, despite the PET results being inconsistent. To mitigate symptoms and confirm the diagnosis, a surgical plan comprising endoscopic transnasal transsphenoidal sellar region exploration, tumor excision, and sellar base reconstruction was devised. At surgery, highly vascularized tumor tissue was encountered, with erosion of the clivus bone. The intraoperative frozen section indicated metastatic HCC, which was further confirmed in postoperative immunohistochemical reports (**Figure 2**). Postoperatively, his headaches and diplopia were significantly alleviated. But unfortunately, his left eye developed ptosis and complete ophthalmoplegia. Postoperative MRI of the brain showed a shrinkage of the lesion (**Figures 1D, E**). He was discharged on post-op day 7 and suggested visiting the oncology clinic for further HCC treatment.

## 3 Results

**Table 1** summarizes all 17 HCC PMs published to date and our case presented here, of which 1 case was included in the surgical series (3) and did not have detailed information. The age of all reviewed patients ranged from 40 to 80, with a median of 65. Only one patient was female.

**Table 1:** Reviewed cases of PMs from hepatocellular carcinoma.

Case No.	Authors	Year	Age	Gender
1	Morita et al.	1998	N/R	N/R
2	Aung et al.	2002	40	M
3	Aung et al.	2002	71	M
4	Karamouzis et al.	2003	59	M
5	Komninos et al.	2004	68	M
6	Hirsch et al.	2005	46	M
7	Chen et al.	2007	68	M
8	Moreno-Perez et al.	2007	65	M
9	Takigawa et al.	2011	58	M
10	Wilson et al.	2013	50	M
11	Tamura et al.	2013	67	M
12	Tamura et al.	2013	58	M
13	He et al.	2015	49	M
14	Shah et al.	2015	65	M
15	Tanaka et al.	2015	80	F
16	Zhang et al.	2019	53	M
17	Ambalavanan et al.	2020	68	M
18	Current	2022	73	M

N/R, not reported.

Their initial clinical manifestations and imaging findings were listed in **Table 2**. Headaches (82.4%) were the most frequent symptoms in HCC PM patients (**Table 3**), followed by diplopia (76.5%), ptosis (52.9%), and visual disturbances (52.9%). Nausea and vomiting (23.5%) and pain in other sites (23.5%) were also seen in a few cases. Some less frequent symptoms included ophthalmoplegia (17.6%), weight loss (17.6%), anorexia (11.8%), polyuria (11.8%), fatigue (11.8%), and weakness (11.8%). Other relatively rare symptoms were not listed, such as bradycardia, decreased libido, hypotension, photophobia, and somnolence.

**Table 2:** Clinical manifestations and imaging characteristics of HCC PM patients.

Case No.	Clinical manifestations	Imaging techniques	Infiltration area
1	N/R	N/R	N/R
2	diplopia, headaches, nausea and vomiting, weight loss	CT, MRI	clivus, petrous apex, sphenoidal sinus
3	headaches, diplopia, ptosis, weight loss	MRI	cavernous sinus, optic chiasma
4	headaches, low back pain, visual disturbances	MRI, PET	optic chiasma
5	headaches, weakness, fatigue, anorexia, somnolence, mild polyuria, ptosis, diplopia, visual disturbances	MRI, cerebral angiography	clivus, sphenoid sinus, suprasellar region
6	headaches, visual disturbances, diplopia, ophthalmoplegia	MRI	cavernous sinus, sphenoidal sinus
7	headaches, ptosis, diplopia	N/R	cavernous sinus, sphenoidal sinus, suprasellar region
8	ptosis, headaches, visual disturbances, diplopia, decreased libido	MRI	cavernous sinus
9	diplopia, visual disturbances	MRI	clivus, optic chiasma, sphenoid sinuses
10	back pain, headaches, diplopia, ptosis, ophthalmoplegia	MRI	cavernous sinus, clivus
11	diplopia, headaches	MRI	clivus, sphenoidal sinus
12	headaches, visual disturbances, fatigue, ptosis, diplopia	CT, MRI, MRA	cavernous sinus
13	visual disturbances, diplopia, headaches, ptosis, ophthalmoplegia, low back pain	MRI	cavernous sinus, clivus
14	headaches, photophobia, diplopia, visual disturbances, ptosis, nausea and vomiting, weight loss	MRI	clivus
15	anorexia, hypotension, bradycardia, diabetes insipidus	MRI	suprasellar region
16	visual disturbances	MRI	suprasellar region
17	headaches, weakness, abdominal pain, nausea and vomiting, ptosis	X-ray, CT	cavernous sinus, maxillary sinus, sphenoidal sinus
18	headaches, diplopia, nausea and vomiting	CT, MRI, PET	cavernous sinus, clivus

**Table 3:** Incidence of different symptoms of HCC PM patients.

Symptoms	Incidence
Headaches	82.4%
Diplopia	76.5%
Ptosis	52.9%
Visual disturbances	52.9%
Nausea and vomiting	23.5%
Pain in other sites	23.5%
Ophthalmoplegia	17.6%
Weight loss	17.6%
Anorexia	11.8%
Diabetes insipidus	11.8%
Fatigue	11.8%
Weakness	11.8%
Others	

The most commonly used imaging technique for diagnosis was MRI (**Table 2**). The metastatic tumors often invaded the cavernous sinus, clivus, and sphenoidal sinus. Infiltrations to optic chiasma, suprasellar cistern, and petrous apex were also reported. X-ray, CT, angiography, and PET were somehow less used.

Concerning the management of HCC PMs patients, surgery was mostly the first option, either by decompressive partial resection or radical resection (**Table 4**). Only two cases took hormone replacement therapy (HRT) treatment. Radiotherapy and chemotherapy were sometimes recommended to those patients as subsequent postoperative management. Two cases underwent transarterial embolism (TAE) because of postoperative nasal hemorrhage. Vascularization was frequently used to describe the tumor lesions encountered at the surgery. The survival time of all cases varied greatly from 2 months to 3 years.

**Table 4:** Management and prognosis of HCC PM patients.

Case No.	Surgery	Other management	Pathology	Survival time
1	N/R	N/R	N/R	N/R
2	sublabial transsphenoidal decompression with partial resection	N/R	soft, grey, vascularized	3-month
3	sublabial transsphenoidal radical resection	HRT, radiotherapy	metastatic HCC	12-month
4	transnasal transsphenoidal decompression with partial resection	HRT	hard, highly vascularized	2-year
5	transsphenoidal decompression	N/R	solid, fibrous, highly vascularized	3-month
6	transsphenoidal exploration and partial resection	radiotherapy, chemotherapy	N/R	12-month
7	N/R	N/R	N/R	4-month
8	transsphenoidal partial resection	N/R	soft, grey	5-month
9	transnasal transsphenoidal decompression with partial resection	TAE	hard, highly vascularized	10-month
10	endoscopic transsphenoidal resection	N/R	N/R	N/R
11	transnasal transsphenoidal partial resection	radiotherapy	hard, vascularized	15-month
12	transnasal transsphenoidal surgery	chemotherapy, radiotherapy, TAE	N/R	5-month
13	endoscopic transnasal transsphenoidal tumor resection	declined	soft, vascularized	3-month
14	transsphenoidal resection using neuronavigation	N/R	soft, pink, friable	N/R
15	none	HRT	N/R	2-month
16	endoscopic transnasal transsphenoidal tumor resection	N/R	hard, vascularized	N/R
17	none	HRT, radiotherapy	N/R	3-year
18	endoscopic transnasal transsphenoidal tumor resection	chemotherapy	soft, highly vascularized	N/R

We further summarized the previous malignancy history of these HCC PMs patients (**Table 5**). A total of 12 patients had previous HCC or hepatitis history. 9 (out of 14) patients had abnormally elevated liver enzymes, and 10 (out of 13) had abnormally elevated AFP.

**Table 5:** Cancer-associated information of HCC PM patients.

Case No.	HCC history	Hepatits history	Liver enzymes	AFP
1	N/R	N/R	N/R	N/R
2	No	HBV	Normal	↑
3	No	HBV	Normal	↑
4	Yes	N/R	Normal	Normal
5	No	N/R	↑	↑
6	No	N/R	↑	↑
7	Yes	HBV	↑	↑
8	No	HCV	↑	Normal
9	N/R	N/R	N/R	N/R
10	N/R	N/R	N/R	↑
11	Yes	HCV	↑	↑
12	No	HBV	Normal	↑
13	No	N/R	↑	↑
14	N/R	HAV, HBV	↑	Normal
15	Yes	HCV	↑	N/R
16	Yes	N/R	N/R	N/R
17	No	HBV	↑	N/R
18	Yes	HBV	Normal	↑

HAV, hepatitis A virus; HBV, hepatitis B virus; HCV, hepatitis C virus.

## 4 Discussion

PMs were relatively rare in pituitary tumors and other intrasellar lesions, with only 1% of all pituitary tumor surgeries reported by retrospective studies (1, 8). However, most metastases were found during the autopsy of malignant patients and the rate could be as high as 28% (2, 5). Most PMs were asymptomatic (9) and the primary malignancy usually deteriorated before metastases-related symptoms occurred (10), which could explain the discrepancy in identification rates between surgical and autopsy series. Clinical characteristics regarding PMs from all cancers were well-documented by previous literature reviews (4, 6). Breast and lung were reported as the most common origins of primary tumor metastatic to the pituitary, which both had been well-reviewed (2). A pooled analysis revealed that a wide spectrum of primary malignancies can metastasize to the pituitary (5).

So far among all reported tumor origins, PMs from HCC were remarkably rare, of which only 16 cases were reported in English literature and 1 case in Chinese literature. Our case presented here became the eighteenth reported. Interestingly, there is a strong male predominance in all reported cases, with only 1 female patient. We interpreted this prevalence as a result of the disproportionate incidences of HCC between males and females (11). According to GLOBOCAN 2018 estimation, the incidence of liver cancer in males was 2.4-fold higher than that in females (7). Another reason is the extremely low frequency of HCC PMs, which only constituted 1.4% of all PMs reported (6).

As mentioned above, most PMs were asymptomatic. In some earlier reviews, the most common clinical presentation reported among symptomatic PMs was diabetes insipidus (DI) (3, 4). But a more recent pooled analysis demonstrated the prevalence of DI was less than visual disturbance, cranial nerve palsies, and anterior pituitary insufficiency. It is believed that the posterior lobe is more likely involved in metastatic cases because its direct arterial blood supply serves as a major spread route (3, 4, 9, 12), leading to the most common symptom of DI. However, controversy emerged when some other studies showed that anterior lobe involvement was more frequent than posterior lobe (13, 14). It is also proposed that the more sensitive immunochemistry test, and imaging techniques as well, largely contributes to the higher detection rate of anterior pituitary insufficiency (6). The incidence of different symptoms in the HCC PMs subpopulation, however, demonstrated a distinct pattern (**Table 3**). The predominant symptoms are headaches and diplopia. Only 2 cases reported polyuria, of which 1 was diagnosed as DI. Other frequently observed symptoms included ptosis and visual disturbances. We hypothesized that the space-occupying effect played an important role in the nervous system involvement of metastatic lesions. As in our case presented, optic chiasm and cavernous sinus were frequently infiltrated or compressed in the reviewed cases (**Table 2**), which is consistent with neurological tests that revealed common defects of optic nerves and cranial nerves III, IV, and VI. Though some investigators believed that the nervous system involvement was underestimated because it may be masked by other HCC-related features, for instance, hepatic encephalopathy (15). Some other symptoms such as nausea, vomiting, and weight loss were possibly related to the primary HCC. Interestingly, only 6 patients were aware of previous malignancy history, and 10 were diagnosed with any type of hepatitis (**Table 5**). Due to the low awareness of primary cancer, it was sometimes misdiagnosed with nonfunctioning invasive macroadenoma (16, 17) and a second operation was performed because of recurrent tumor enlargement.

Diagnosing PMs remains difficult, especially among patients with unknown malignant diseases (18). One obstacle is the differentiation between PM and adenoma, for their clinical presentations and radiology characteristics are indistinguishable. Some investigators suggested that bony erosion without sellar enlargement indicated metastases (19). Bony erosion was also observed in our case. The diagnosis of HCC PMs has the same predicament. The most commonly used diagnostic imaging technique is MRI, in which a mass was often recognized within the sellar region. However, no diagnostic pattern can be identified. There are also attempts to use PET (20) for metastases identification, as PET scan performs better than conventional imaging techniques in revealing metastases (21). However, reports indicate that benign lesions such as adenoma can manifest as hypermetabolic loci (22). In our case, multiple hypermetabolic loci were observed in the liver and bone, while the sellar region exhibited no elevated radiation uptake. We hypothesized that it is the result of the variable uptake exhibited by primary HCC (23), or the elevation, if any, was masked by brain parenchyma background uptake. Chordoma (24) and chondrosarcoma (25) were taken into consideration for their typical clivus involvement. Diagnosis can be confirmed only by intraoperative frozen section and postoperative histopathology. In our case, we encountered highly vascularized tumor tissue at surgery, which was consistent with previous studies (26, 27). Primary hepatocellular carcinoma biopsy was not performed in our case. According to the 2019 WHO classification, HCC can be divided into several subtypes based on their molecular characteristics (28). In 17 cases reviewed, only half (8) performed liver biopsy, of which 4 reported pathological patterns. Two of them were pseudoglandular and the other two were solid and acinar. Certain subtypes exhibit distinct behaviors and prognoses, and they can be indicative of the development of HCC PMs.

Surgery is still the priority for the management of PMs, serving as a palliative treatment to improve life quality. Endocrinological replacement is necessary for pituitary insufficiency. Other available options include radiotherapy, chemotherapy, and other modalities (6). Decompressive tumor resection was usually the first option in HCC PMs patients (**Table 3**). Radical resection is rarely operated (29). Additionally, radiotherapy and chemotherapy are recommended together or individually as subsequent postoperative management. Surgery largely alleviates symptoms in the cases reviewed, including headaches, visual defects, ophthalmoplegia, and so on. But there is not always space for surgical treatment because metastatic lesions tend to be invasive, diffuse, and highly vascularized as mentioned before (30, 31), or the general condition of the patient does not tolerate surgery (32).

The prognosis does not depend on the manifestation or the management of metastases (31). No statistical difference in survival time was observed between surgical and nonsurgical groups (3). It is rational because metastases generally indicate an advanced stage in the development of malignancies and multiple systems were likely affected at that time. Therefore, treatments aiming at the primary malignancy should be highlighted. First-line recommendations by American Gastroenterological Association (AGA) for metastatic HCC include locoregional therapies (LRTs) and systemic therapy (33). Transarterial chemoembolization (TACE) and transarterial radioembolization (TARE) are commonly adopted LRTs. There are two successful reports of TACE application to control active nasal bleeding postoperatively (34, 35), which implies a potential application of endovascular management in PMs. Sorafenib, a tyrosine kinase inhibitor (TKI), is the first drug approved for HCC systemic therapy. Recently, more systemic options have arisen and proven effective in multiple clinical trials. For example, the combination of atezolizumab and bevacizumab is recommended as the first-line treatment in advanced or metastatic patients with preserved liver function (33). Other promising drugs include regorafenib and cabozantinib, which are preferred in refractory patients (36). However, we did not see any proper application of systemic therapy in reviewed cases. One important reason is that many cases were reported before 2007 when sorafenib was firstly approved. We believed that with more cases reported, the application of systemic therapy will be proven to improve the prognosis and life quality of patients with PMs.

## 5 Conclusion

We presented a rare case of HCC PMs in a 73-year-old man presented with headaches and diplopia. He had hepatitis and HCC history, and recent imaging showed primary cancer recurrence and metastases. Endoscopic excision was performed to alleviate his symptoms and assist diagnosis. Literature review contains 17 documented cases. Consensus about the diagnosis and management is challenging due to the low incidence. Headaches and diplopia are typical initial symptoms. A majority of these patients have known history of HCC or hepatitis infection. Therefore, we recommended taking further examinations with patients who have sudden-onset headaches and previous HCC-related history. Surgical excision is recommended to ameliorate symptoms and improve life quality.

## Author contributions

Both authors have made substantial contributions to the conception of this work. YC drafted the first version of the manuscript. MF revised the manuscript and provide approval for publication. All authors contributed to the article and approved the submitted version.
